# Detection and Characterization of Hepatitis E Virus in Goats at Slaughterhouse in Tai'an Region, China

**DOI:** 10.1155/2017/3723650

**Published:** 2017-12-12

**Authors:** Song Li, Mingxia Liu, Jingjing Cong, Yufa Zhou, Zengmin Miao

**Affiliations:** ^1^College of Basic Medicine, Taishan Medical University, Tai'an 271000, China; ^2^College of Life Sciences, Taishan Medical University, Tai'an 271000, China; ^3^Center for Disease Control, Veterinary Bureau of Daiyue, Tai'an 271000, China

## Abstract

**Background:**

Hepatitis E virus (HEV) is a significant pathogen of viral hepatitis and can be transmitted through fecal-oral route. Epidemiological data concerning HEV in goats, however, are relatively sparse to date. Here, the prevalence and characteristics of HEV isolated from goats at slaughterhouse were investigated in Tai'an region, China.

**Methods:**

Anti-HEV immunoglobulin G (IgG) in blood samples and HEV RNA in the liver samples were determined by using an enzyme-linked immunosorbent assay (ELISA) and a nested reverse transcription polymerase chain reaction (RT-PCR), respectively. In addition, partial nucleotide sequences of open reading frame 2 (ORF-2) of HEV isolates were analyzed.

**Results:**

Fifty goat blood samples (46.7%, 50/120) were masculine for anti-HEV IgG. HEV RNA was detected in 2 liver samples (4.0%, 2/50) and belonged to genotype 4 subtype 4 h, with high identity (91.2–93%) with cow HEV strains detected in the same province, China.

**Conclusions:**

These findings demonstrated that goats may be an important reservoir for HEV and can become a major source of HEV infection in humans via food chain.

## 1. Introduction 

Hepatitis E virus (HEV) belonging to the Hepeviridae family is the only member of the* Orthohepevirus* genus and is a nonenveloped, positive-sense, single-stranded RNA virus [1, 2]. Although HEV infection in humans is sporadic in industrialized countries [3–6], the infection is frequently detected in many developing countries [7, 8]. A majority of HEV infections are asymptomatic and self-limiting, but infections with acute liver failure can develop chronic hepatitis among immunosuppressed persons [9, 10].

To date, HEV can be categorized into at least 7 genotypes [11, 12]. Genotype 1 and 2 HEVs are known to only infect humans and are associated with most HEV outbreaks and endemics in developing countries [11, 13, 14]. Genotypes 3 and 4 have been frequently isolated from humans and animals [15–18]. Genotype 3 is globally prevalent, but genotype 4 is mainly prevalent in Asian and European countries [19–23]. HEV isolates from wild boar in Japan are regarded as genotypes 5 and 6 [24]. HEV isolated from camels in the Middle East belongs to genotype 7 [25].

In addition to the fecal-oral route [1, 2], consumption of contaminated water [26, 27], raw or undercooked animal products or inner organs [28–30] could lead to HEV infection. Goats are generally raised as an economically important source of meat and milk in Tai'an region of China, in which mixed farming practice of cows and goats is used by farmers. However, until now little information is available on HEV infection data in goats. Therefore, this study was carried out to investigate the prevalence and molecular characterization of HEV isolates from goats at slaughterhouse in Tai'an region, China, with the aim of providing epidemiological data for HEV infection.

## 2. Materials and Methods

### 2.1. Sample Collection

One hundred and twenty blood samples and 50 liver samples were randomly collected from healthy adult goats (2-3 years old) at a slaughterhouse in Tai'an region, China, between January and March 2017. Goats at the slaughterhouse were bought from farmers in the surrounding villages. Generally, this region adopts traditional mixed farming of cows and goats. Typically, each household raises one to three cows and five to twenty goats. Sampling procedures were approved by the Taishan Medical University Animal Care and Use Committee (permit number: 20170117).

### 2.2. Detection of Anti-HEV Antibody

According to previous reference [31], a commercial enzyme-linked immunosorbent assay (ELISA) kit (Peking Dingguo, China) specific to goat's anti-HEV immunological G (IgG) was used to test the blood samples, based on the manufacturer's instructions.

### 2.3. Extraction of RNA

Approximately 20 mg of each liver sample was homogenized with phosphate-buffered saline on ice, and the suspension was then centrifuged at 12,000 ×g for 10 min at 4°C. Total RNA was extracted from the supernatant using TRIZOL reagent (Invitrogen, USA).

### 2.4. Nested RT-PCR

According to protocols and primers previously described [16, 31, 32], a nested reverse transcription polymerase chain reaction (RT-PCR) was conducted to amplify partial nucleotide sequences (348 nt) of open reading frame 2 (ORF-2) of HEV. For the first round of PCR, the primers were HEV-1 [forward primer: 5′-AATTATGCC(T)CAGTAC(T)CGG(A)GTTG-3′] and HEV-2 [reverse primer: 5′-CCCTTA(G)TCC(T)TGCTGA(C)GCATTCTC-3′], and HEV-3 [forward primer: 5′-GTT(A)ATGCTT(C)TGCATA(T)CATGGCT-3′] and HEV4 [reverse primer: 5′-AGCCGACGAAATCAATTCTGTC-3′] were used for the second round. HEV RNA negative liver samples were all retested by the nested RT-PCR. The products of the nested RT-PCR were analyzed in a 1.5% agarose gel.

### 2.5. Nucleotide Sequencing

The expected DNA band was purified by using a DNA Gel Extraction kit (Axygen, USA). The purified products were cloned into pMD T-Vector (TaKaRa, Japan). A DNA analyzer (Applied Biosystems 3730 DNA Analyzer, Invitrogen) was employed to sequence the inserted DNA segment.

### 2.6. Phylogenetic Analysis

The 2 HEV isolates in this study and different genotypes of HEV strains were aligned using MEGA software 5.0 version. The standard definition of HEV genotypes was performed based on previous studies [33, 34]. The neighbor-joining method was used to construct a phylogenetic tree. The MegAlign program (DNAstar package, version 5.03) was used to analyze the identity between nucleotide sequences.

## 3. Results

### 3.1. Seroprevalence of Anti-HEV Antibody and HEV RNA

Out of 120 goat blood samples, 50 samples were positive for anti-HEV antibody (46.7%, 50/120). Among 50 raw goat livers tested for HEV RNA, 2 samples (4.0%, 2/50) were found positive.

### 3.2. Phylogenetic Analysis of HEV Isolated from Goats

**Figure 1:**
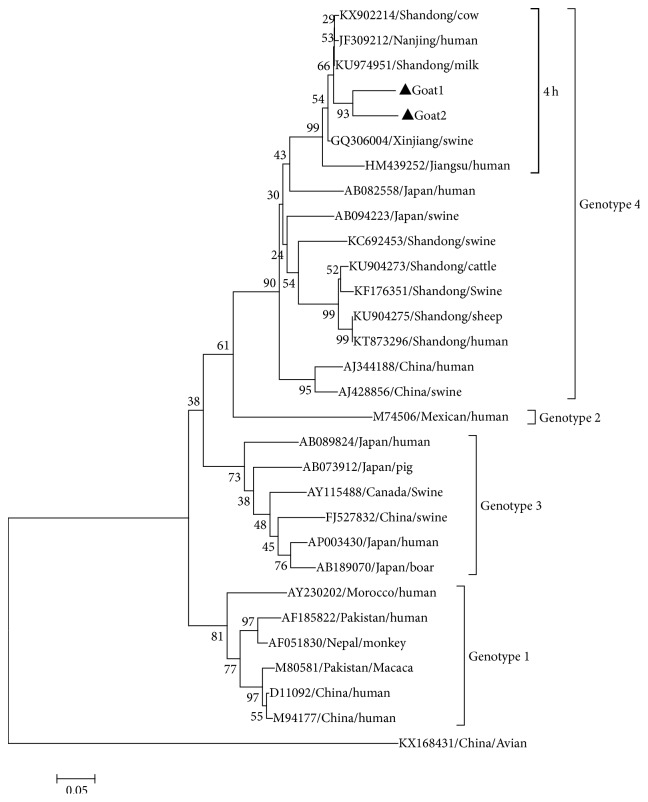
Phylogenetic tree constructed by alignment of the 348 nt nucleotide sequence of ORF2 of HEV isolates in this study and 27 HEV reference isolates from animals and humans.* Note*. An avian HEV strain is included as outgroup and 2 HEV isolates in this study were marked with black triangle.

Phylogenetic analysis based on HEV ORF-2 partial sequences (348 nt) demonstrated that the 2 HEV strains in this study belonged to HEV genotype 4 subtype 4 h (Figure 1). The 2 isolates designated as Goat 1 and Goat 2 (GenBank accession numbers: MF443448 and MF443449) shared 89.9% nucleotide sequence identity. The two HEV strains shared up to 92.5%–93% sequence identity with those previously isolated from cows in 2016 in the same province of China (KU974951, KX902214). In addition, the two HEV strains had 75.6%–81.8% genetic similarity with the HEV isolated from humans in Jiangsu province of China (JF309212, HM439252) and 75.3%–76.6% similarity with the HEV isolated from pigs in Xinjiang region of China (GQ306004) (Table 1).

## 4. Discussion

In the present study, anti-HEV IgG was positive in 50 goat serum samples (46.7%, 50/120) and HEV RNA was identified in 2 liver samples (4.0%, 2/50). Parallel result was found in Yunnan province of China: 67% of serum samples were masculine for anti-HEV IgG in goats and HEV RNA was found in the feces, serum, and milk samples [35]. In addition, HEV RNA was detected in goats in Teramo (9.24%), Italy [36]. These findings indicated that goats may be an important reservoir for HEV.

Up to date, several studies have provided evidence that HEV can be transmitted to humans or nonhuman primate via close contact with animals or through consumption of raw or undercooked meats and milk [37–39]. It is noteworthy that HEV RNA was detected in the goat livers in this study, which may enhance transmission risk of HEV through food chain because consumption of meat, milk, and inner organs of goats are popular in Tai'an region, China.

Phylogenetic analysis showed that the two HEV isolates in this study belonged to 4 h subtype of genotype 4 and shared a high similarity with the cow HEV (92.5%–93%) isolated in the same province of China in 2016 (KU974951, KX902214), which indicated that cross-species transmission of HEV between domestic animals may occur. It is noteworthy that the result may be associated with the mixed farming practice used by the local farmers for raising cows and goats in the backyard. It is interesting to note that the goat HEV isolated in Italy belonged to genotype 3 [36], but the goat HEV isolated in this study was of genotype 4. This difference in HEV genotype in the same host may be related to the geographical difference.

There are some limitations in this study; for example, other samples of goat detected, such as feces, sera, and milk, were not detected, histopathological and immunohistochemical examinations in these HEV RNA positive goat livers was not performed, and the sample size was relatively small. But our present study to some extent reflected the prevalence of HEV in goat at slaughterhouse and further confirmed that goats may be an important reservoir for HEV. Of note, high nucleotide sequence identity of HEV isolates was found between the goats and cows within the same province of China, indicating that there may be cross-species transmission of HEV among domestic animals.

## Figures and Tables

**Table 1:** Sequence identity (%) among 7 HEV strains (subtype 4 h, genotype 4), including the 2 goat liver HEV isolates and 5 HEV reference strains.

Sequences	Swine (1)	Human (2)	Cow (2)	Goat 2
Goat 1	76.6	76.9–81.8	92.5–93.0	89.9
Goat 2	75.3	75.6–80.5	91.2–91.7	
Cow (2)	82.3	82.6–87.3		
Human (2)	84.9–96.9			

*Note*. Numbers in parentheses stand for number of HEV reference sequences: swine (GQ306004); human (JF309212, HM439252); cow (KU974951, KX902214).
